# Oncolytic effect of wild-type Newcastle disease virus isolates in cancer cell lines in vitro and in vivo on xenograft model

**DOI:** 10.1371/journal.pone.0195425

**Published:** 2018-04-05

**Authors:** Kseniya S. Yurchenko, Peipei Zhou, Anna V. Kovner, Evgenii L. Zavjalov, Lidiya V. Shestopalova, Alexander M. Shestopalov

**Affiliations:** 1 Research Institute of Experimental and Clinical Medicine–subdivision of FRC FTM, Novosibirsk, Novosibirsk Region, Russia; 2 Novosibirsk State University, Novosibirsk, Novosibirsk Region, Russia; 3 Heilongjiang University, Harbin, Heilongjiang Sheng, China; 4 Center for Genetic Resources of Laboratory Animals, Institute of Cytology and Genetics, Siberian Branch of the Russian Academy of Sciences, Novosibirsk, Novosibirsk Region, Russia; Northwestren University, UNITED STATES

## Abstract

Oncolyic virotherapy is one of the modern experimental techniques to treat human cancers. Here we studied the antitumor activity of wild-type Newcastle disease virus (NDV) isolates from Russian migratory birds. We showed that NDV could selectively kill malignant cells without affecting healthy cells. We evaluated the oncolytic effect of 44 NDV isolates in 4 histogenetically different human cell lines (HCT116, HeLa, A549, MCF7). The safety of the isolates was also tested in normal peripheral blood mononuclear (PBMC) cells. The viability of tumor cell lines after incubation with NDV isolates was evaluated by MTT. All cell lines, except for normal PBMC primary cells, had different degrees of susceptibility to NDV infection. Seven NDV strains had the highest oncolytic activity, and some NDV strains demonstrated oncolytic selectivity for different cell lines. In vivo, we described the intratumoral activity of NDV/Altai/pigeon/770/2011 against subcutaneous non-small cell lung carcinoma using xenograft SCID mice model. All animals were responsive to therapy. Histology confirmed therapy-induced destructive changes and growing necrotic bulk density in tumor tissue. Our findings indicate that wild-type NDV strains selectively kill tumor cells with no effect on healthy PBMC cells, and intratumoral virotherapy with NDV suppresses the subcutaneous tumor growth in SCID mice.

## Introduction

Newcastle disease virus (NDV), or avian paramyxovirus serotype 1 (APMV-1), which belongs to the family *Paramyxoviridae* [1, 2], causes severe Newcastle disease in poultry and wild birds worldwide [3, 4]. However, NDV is non-pathogenic for mammals and therefore represents a promising virotherapeutic agent for human malignancies [5].

The oncolytic activity of NDV has been investigated since 1952 [6], and National Institute of Oncology (NCI) included NDV in the list of complementary and alternative therapies [7]. IFN response prevents NDV replication in healthy cells [8–11]. However, NDV uses uncontrolled division and the mobilized synthetic apparatus of cancer cells with aberrant IFN-response to produce viral progeny and induce oncolysis [12]. Several NDV strains have proved efficient *in vitro*, *in vivo* and in phases I and II of clinical trials [5, 13–20].

The current trend is to use recombinant strains with decreased pathogenicity and improved antitumor effect [21–26]. However, naturally occurring oncolytic NDV strains are also observed.

The oncolytic potential of NDV strains circulating in wild migratory birds of Russia remains poorly understood. Here we describe oncolytic wild-type NDVs from natural reservoirs obtained in 2008–2014 in Russia. We report the rejection of excessive attenuations and the usage naturally occurring NDV strains. Oncolytic properties were determined using 4 tumor cell lines of various histogenesis. We demonstrate the *in vitro* ability of NDVs to influence the viability of tumor cells after infection and evaluate in vivo efficiency of NDV strain against non-small cell lung carcinoma.

## Results

### Viruses

Newcastle disease virus was isolated from wild migratory birds in eight administrative regions of the Russian Federation: the Altai Territory, the Novosibirsk Region (Western Siberia), the Republic of Tyva (Eastern Siberia), the Amur Region, the Kamchatka Territory, the Republic of Sakha (Yakutia), the Sakhalin Region (Far East) and the Republic of Adygea (Southern Federal District). A total of 44 wild-type NDV isolates were collected in Siberia and the Far East of the Russian Federation in 2008–2014.

### Cytotoxicity of NDV strains in human peripheral blood mononuclear cells (PBMC)

**Fig 1:**
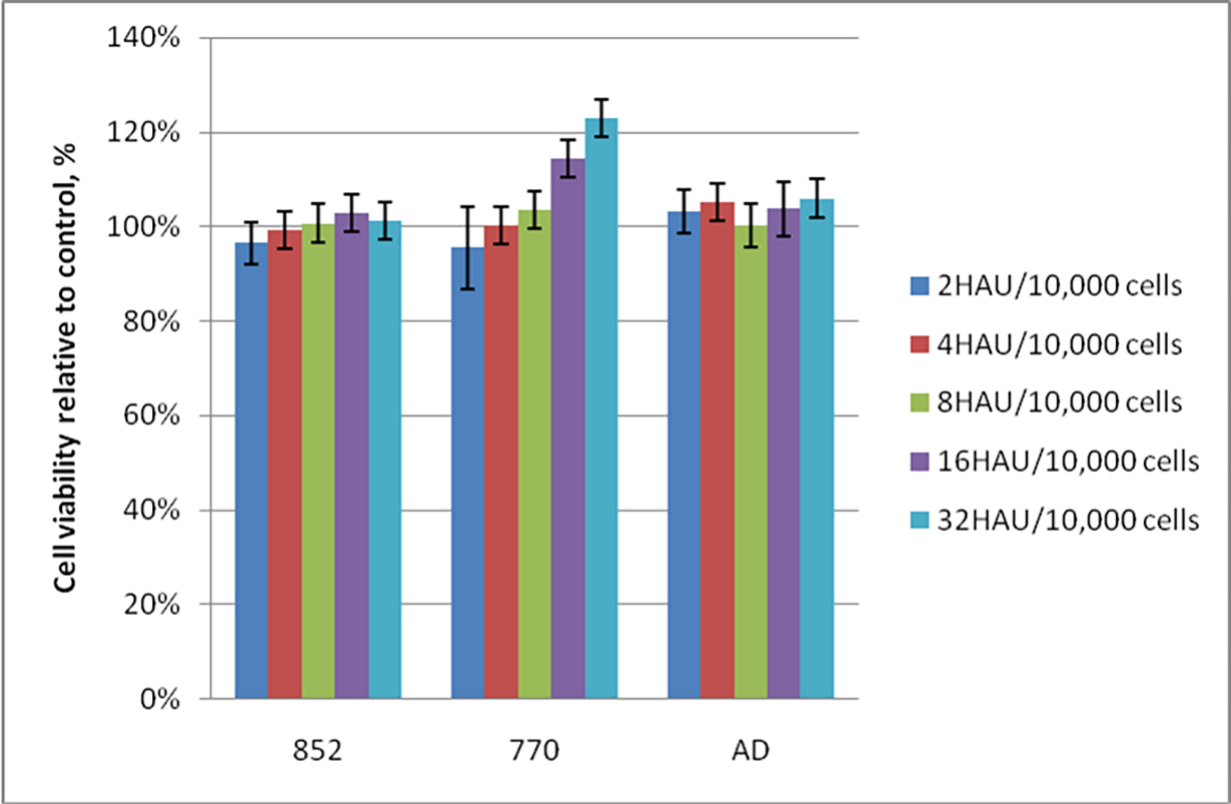
The viability of human peripheral blood mononuclear cells after NDV infection. The viability of the normal human PBMC cell line after incubation with wild-type NDV strains, 4^th^ day after viral infection. The MTT results of cells incubated with fresh medium were taken as a control (100%).

The selected strains represented different NDV pathotypes: NDV/Yakutiya/mallard/852/2011 (852)–mesogenic pathotype with the typical avirulent type F-gene sequence [27], NDV/Altai/pigeon/770/2011 (770)–mesogenic pathotype with the typical virulent type F-gene sequence [28] and Adygea/duck/12/2008 (AD)–velogenic pathotype [29]. There were no changes in viability of suspended PBMCs even after 4 days of infection with different NDV strains. The viability ranged from 94% to 110% of that of controls. NDV-infected PBMC cell culture had no visible morphological disorders compared to controls after an hour of viral exposure and on the following days of cultivation. MTT assay also shows that the strains have no toxic effect on PBMCs because of unchanged cell viability after infection (Fig 1). Thus, we demonstrated that viral strains were safe for human cells regardless of pathotype.

### *In vitro* cytotoxicity

**Fig 2:**
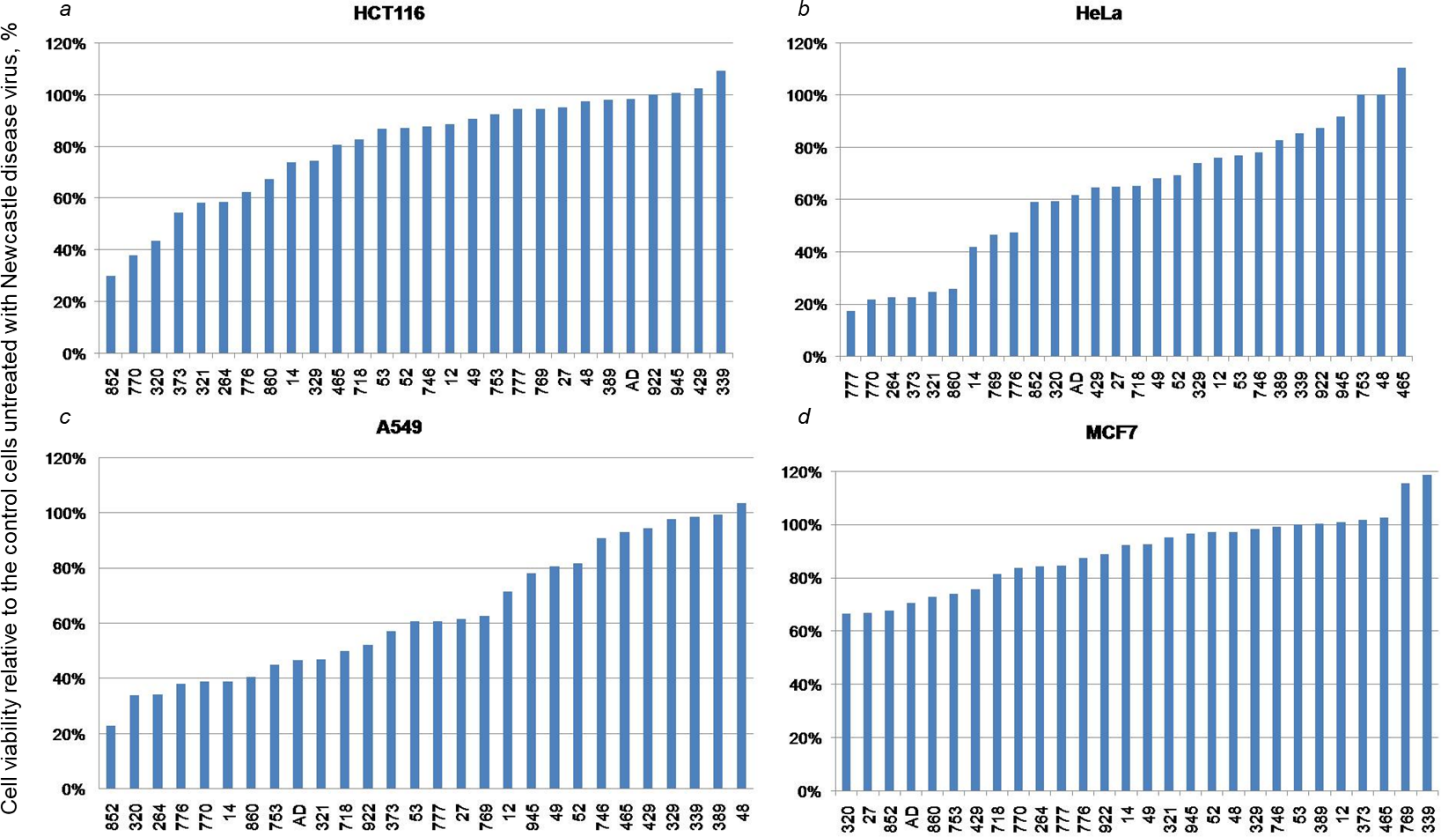
The viability of human tumor cell lines after NDV-infection. The viability of (a) HCT116, (b) HeLa, (c) A549 and (d) MCF7 cell lines after incubation with wild-type NDV isolates, 4^th^ day after viral infection. The MTT results of cells incubated with fresh medium were taken as control (100%).

The cytotoxic properties of Newcastle disease virus isolates were assessed using MTT assay at 540 nm in four tumor cell lines: A549, MCF7, HeLa and HCT116. The MTT assay gives an idea of the metabolic activity of the cells being studied, which allows one to estimate the specific cell death after infection with the virus. Cells were grown on plates for one day and infected with viral dilutions of 2, 8 and 16 HAU per 10.000 cells. The assay was performed on 4^th^ day after cell monolayer infection. Fig 2 show MTT assay results for HCT116, HeLa, A549 and MCF7, respectively.

#### The cytotoxic effect of NDV isolates on the HCT116 cell line (human colorectal cancer)

Fig 2A shows more pronounced cytotoxic effect of NDV infection in HCT116 cells compared with PBMC. HCT116 cell death ratio varied depending on the strain. Despite the cytotoxic effect, HCT116 cells retained high viability (≥80%) after infection with 60% of studied NDVs, and 25% of isolates did not affect the proliferative activity of the cells, based on MTT assay.

**Fig 3:**
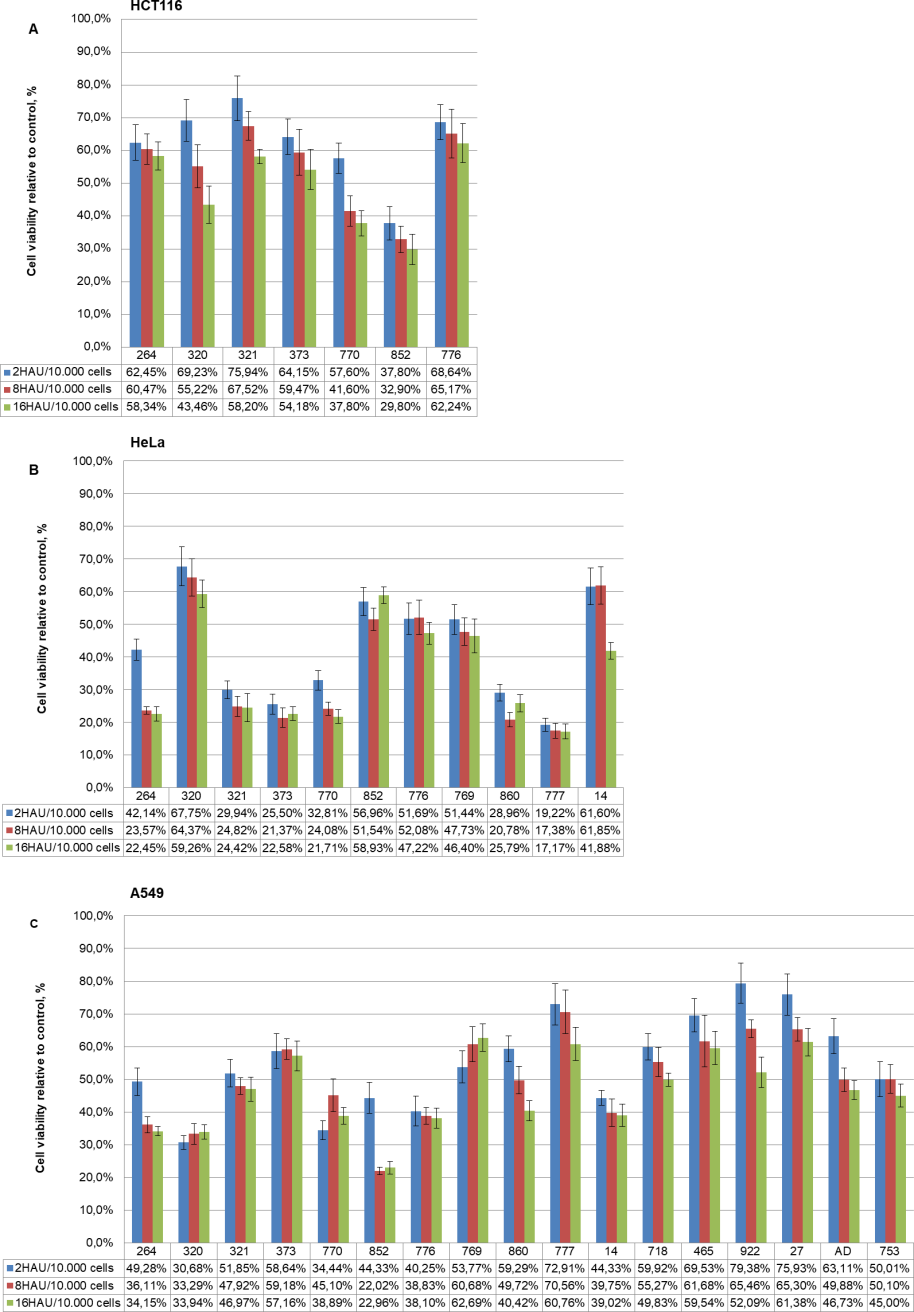
The oncolytic effect of NDV isolates on human tumor cell line in vitro. (A) Colorectal cancer HCT116 cells, (B) cervical cancer HeLa cells, (C) non-small cell lung cancer A549 cells. The MTT analysis on the 4^th^ day after infection (2, 8 and 16 HAU per 10.000 cells).

The following NDV strains had the most pronounced oncolytic effect on the 4^th^ day after infection with a dose of 16 HAU per 10,000 cells: NDV/Yakytiya/mallard/852/2011 (29.80% ± 4.60% of viable tumor cells), NDV/Altai/pigeon/770/2011 (37.80% ± 3.85% of viable tumor cells) and NDV/teal/Novosibirsk region/320/2010 (43.46% ± 5.65% of viable tumor cells). The viability of cells infected with these strains was two times lower than that of control cells. The oncolytic effects of other isolates was less pronounced with the viability ranging from 50% to 60% on the 4^th^ day after infection with a dose of 16 HAU per 10,000 cells: NDV/Novosibirsk/garganey/373/2010 (54.18% ± 6.12%), NDV/Novosibirsk/garganey/321/2010 (58.20% ± 2.14%), NDV/mallard/Amur/264/2009 (58.34% ± 4.36%) and NDV/Novosibirsk/shoveler/776/2010 (62.24% ± 5.96%) (Fig 3A).

#### The cytotoxic effect of NDV isolates on HeLa cell line (human epidermoid cervical carcinoma cells)

Human cancer HeLa cells demonstrated the oncolytic effect of 11 NDV isolates, including seven mentioned above and four additional: NDV/Novosibirsk/garganey/769/2008, NDV/Yakutiya/mallard/860/2009, NDV/Altai/Pigeon/777/2010 and NDV/Tyva/gull/14/2014 (Fig 2B).

The following NDV strains had the most pronounced oncolytic effect (less than 30% of viable cells) on the 4^th^ day after infection with a dose of 16 HAU per 10,000 cells: NDV/Altai/pigeon/777/2010 (17.17% ± 2.36%), NDV/Altai/pigeon/770/2011 (21.71% ± 2.10%), NDV/mallard/Amur/264/2009 (22.45% ± 2.19%), NDV/Novosibirsk/garganey/373/2010 (22.58% ± 2.12%), NDV/Novosibirsk/garganey/321/2010 (24.42% ± 4.32%), NDV/Yakutiya/mallard/860/2009 (25.79% ± 2.70%) (Fig 3B).

#### The cytotoxic effect of NDV isolates on A549 cell line (human non-small cell lung cancer)

A total of 17 NDV isolates effectively eliminated tumor cells (up to 60% of tumor cells remained viable). These isolates included the above mentioned 11 and 6 additional isolates: NDV/Novosibirsk/mallard/718/2008, NDV/Sakhalin/pintail/53/2010, NDV/Amur/garganey/922/2010, NDV/Novosibirsk/garganey/27/2014, NDV/Adygea/duck/12/2008, NDV/Novosibirsk/garganey/753/2008 (Fig 2C).

**Fig 4:**
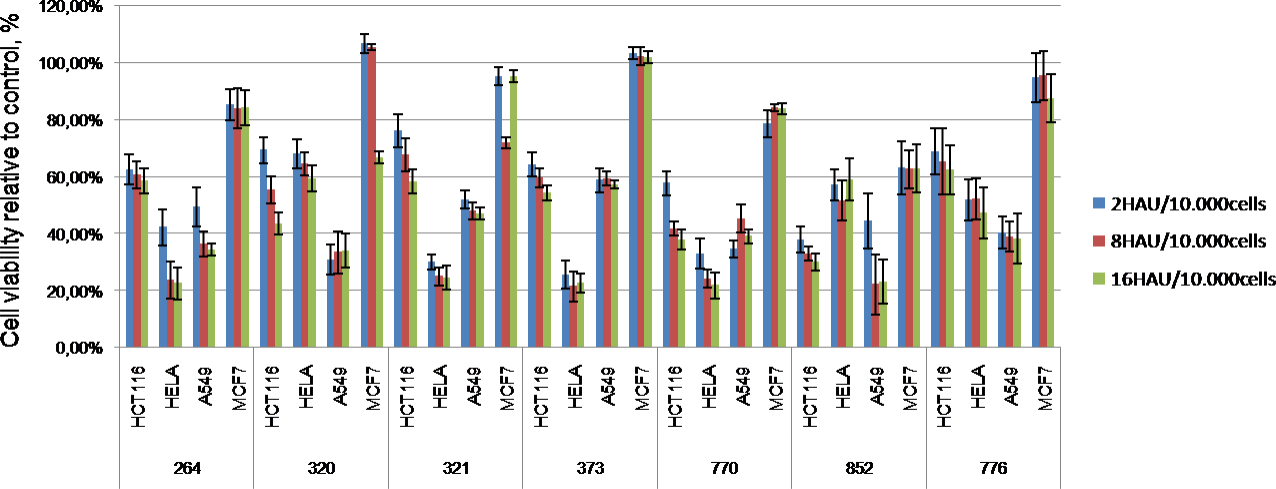
Wild-type NDV isolates with the most pronounced oncolytic properties against all studied human tumor cell lines in vitro. MTT analysis, 4^th^ day after infection (2, 8 and 16 HAU per 10.000 cells).

The following strains had the most pronounced antitumor effect (up to 40% of viable cells after infection): NDV/Yakytiya/mallard/852/2011 (22.96% ± 1.95%), NDV/teal/Novosibirsk region/320/2010 (33.94% ± 2.17%), NDV/mallard/Amur/264/2009 (34.15% ± 1.34%), NDV/Novosibirsk/shoveler/776/2010 (38.10% ± 3.01%), NDV/Altai/pigeon/770/2011 (38.89% ± 2.54%) and NDV/Tyva/gull/14/2014 (39.02% ± 3.43%) (Figs 3C and 4).

#### The cytotoxic effect of NDV isolates on MCF7 cell line (human breast adenocarcinoma)

Human mammary adenocarcinoma cells MCF7 proved the most resistant to all NDV isolates. MTT assay showed a relatively small decrease in cell viability after incubation with viruses (from 80% to 100% of cells remained viable) (Fig 2D).

The following isolates showed the greatest antitumor activity in MCF7 cells on the 4^th^ day after infection with a dose of 16 HAU per 10,000 cells: NDV/Teal/Novosibirsk region/320/2010 (66.58%±7.65% of viable cells), NDV/Novosibirsk/garganey/27/2014 (66.82%±6.53%), NDV/Yakutiya/mallard/852/2011 (67.70%±8.73%), NDV/Adygea/duck/12/2008 (70.60%±6.47%), NDV/Yakutiya/mallard/860/2009 (72.74%±6.54%), NDV/Novosibirsk/garganey/753/2008 (73.89%±6.26%), NDV/Novosibirsk/shoveler/429/2010 (75.51%±6.45%), NDV/Novosibirsk/mallard/718/2008 (81.35%±7.45%), NDV/Altay/pigeon/770/2011 (83.80%±8.96%).

### *In vivo* virotherapy

NDV/Altai/pigeon/770/2011 was chosen for virotherapy as a strain with the highest oncolytic activity in vitro and mesogenic pathotype. Previous studies suggest that more virulent NDV strains may have improved oncolytic efficacy [30], which makes NDV/Altai/pigeon/770/2011 a promising agent.

Cytotoxicity experiments with tumor cell lines demonstrated a wide range of cell susceptibility to NDV infection. The number of viable non-small-cell lung carcinoma A549 cells decreased to less than 40% after NDV/Altai/pigeon/770/2011 infection, according in vitro test. Here we hypothesize that mesogenic strain may have high anticancer efficiency in vivo in a xenograft model of A549 cells.

To determine the efficacy of NDV virotherapy in vivo, SCID mice were injected subcutaneously with 3.5 × 10^6^ A549 tumor cells. After 20 days, when an average tumor diameter reached 4 mm, tumor-bearing mice received a course of intratumoral injections of NDV/Altai/pigeon/770/2011 during 4 days. Control mice received a series of PBS injections during 4 days.

#### Therapeutic efficacy of pigeon NDV strain in tumor-bearing mice

**Fig 5:**
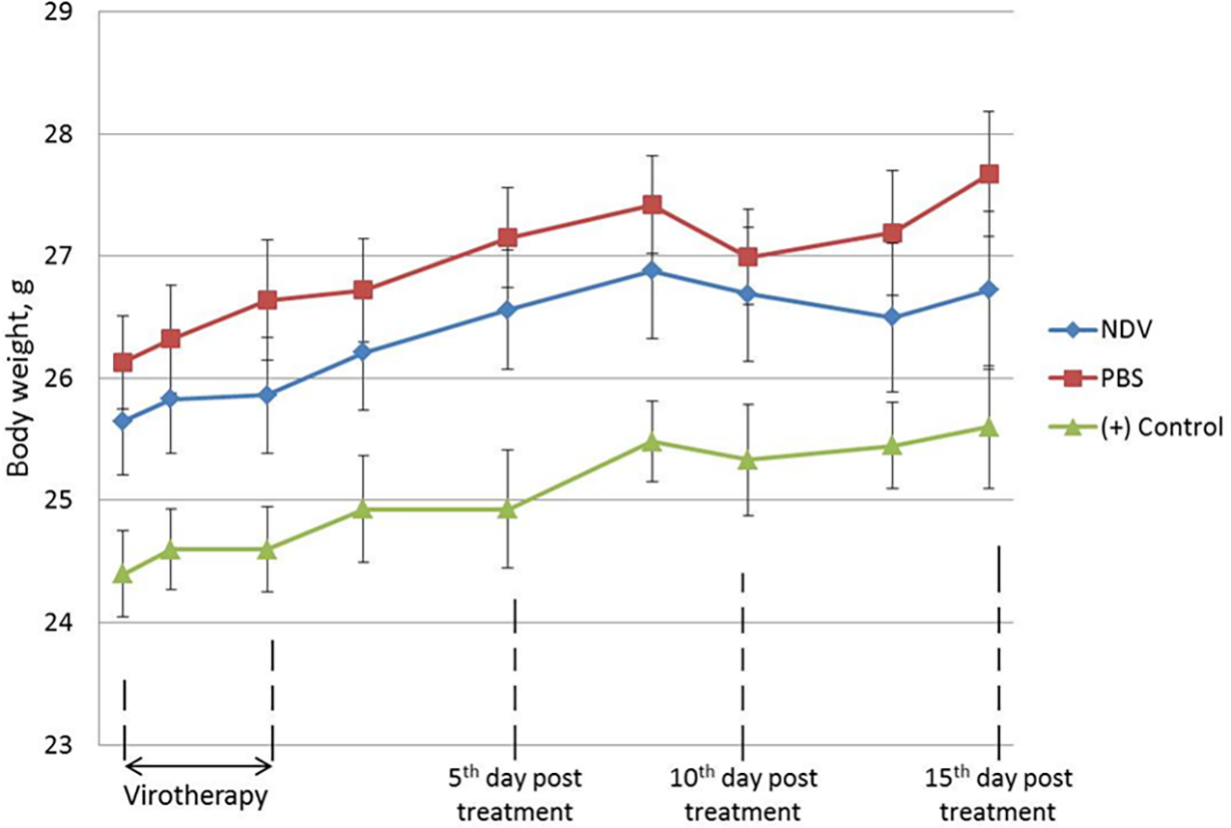
The weight dynamics of SCID non-small cell lung cancer-bearing mice after infection with NDV. Tumor-bearing mice intratumorally infected with NDV/Altai/pigeon/770/2011 (NDV) and mice intratumorally injected with phosphate- buffered saline (PBS). (+) Control is a group of animals without tumors that received subcutaneous injections of NDV/Altai/pigeon/770/2011. The zero time point indicates the initiation of therapy. Weight dynamics is graphically shown as average relative values with standard errors (mean relative value ± standard deviation, M±SE).

**Fig 6:**
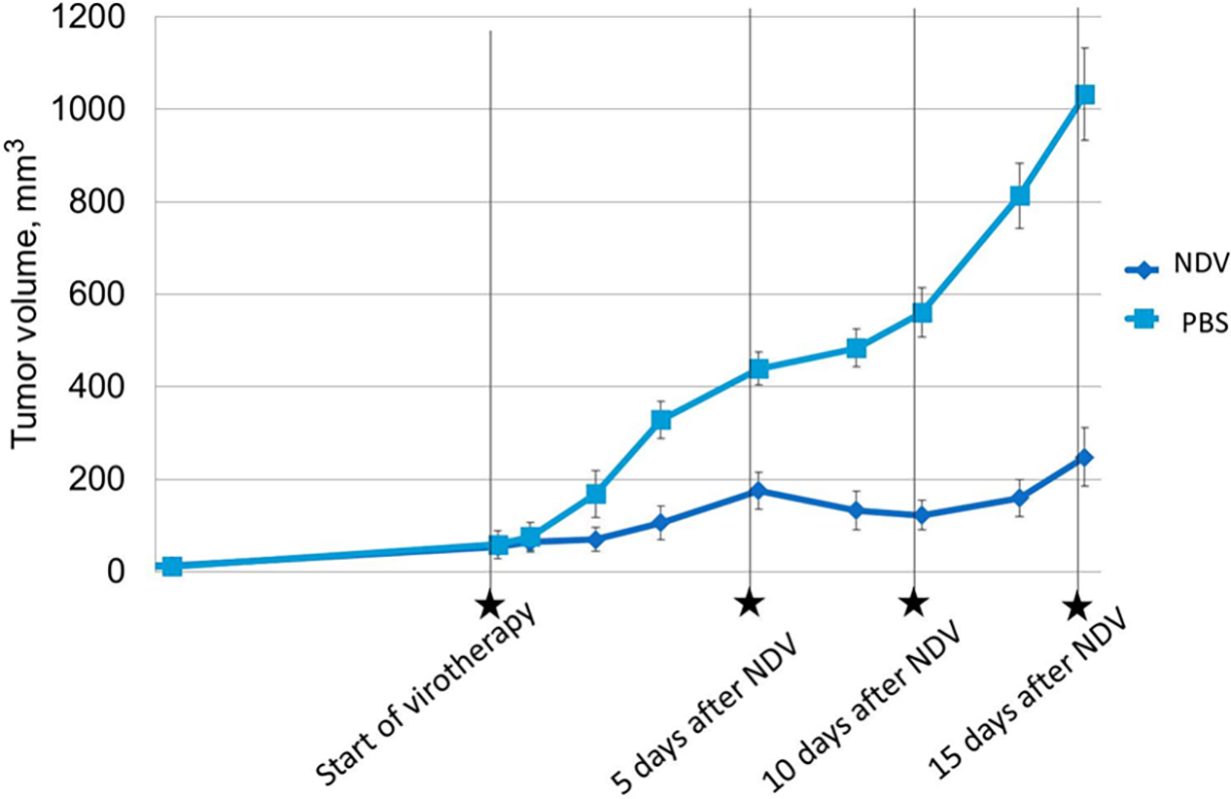
Tumor growth of SCID non-small-cell lung cancer-bearing mice after infection with NDV. Tumor-bearing mice intratumorally infected with NDV/Altai/pigeon/770/2011 (NDV) and mice intratumorally injected with phosphate- buffered saline (PBS) (four injections total). The zero time point indicates the initiation of therapy. Dynamics is graphically shown as the average relative values with standard errors (mean relative value ± standard deviation, M±SE).

A549 tumor-bearing mice were infected with NDV/Altai/pigeon/770/2011 when tumor size reached 4 mm in diameter. Fig 5 shows that weight dynamics was the same in three groups of mice and controls (+) with an increase and a slight decrease of body weight. There was therefore no effect of viral injections on weight dynamics. Controls (+) had a lower weight due to the absence of tumors. Tumor nodules in PBS group were larger than those of virotherapy group (Fig 6).

**Fig 7:**
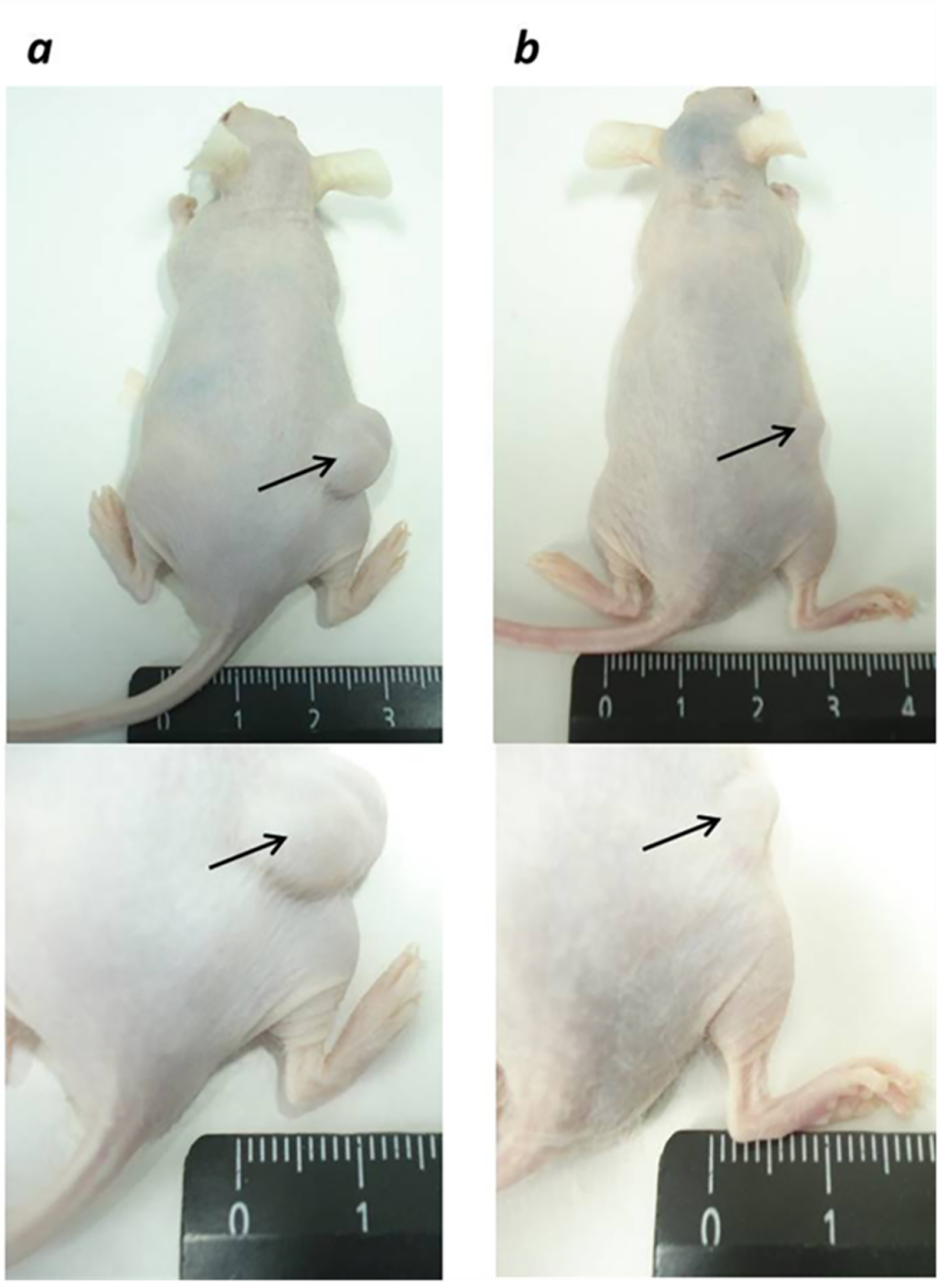
*In vivo* NDV therapy resulted in inhibition of subcutaneous tumor nodes of non-small-cell lung A549 carcinoma-bearing SCID mice. (a) Mice from control group receiving PBS injections. (b) Mice intratumorally injected with four doses of NDV/Altai/pigeon/770/2011 (7 lgTCID50/100μl) during 4 days. Areas with tumor nodules are denoted by arrows. 15^th^ day after virotherapy (38^th^ day of tumor growth).

On 20^th^ day (after A549 tumor inoculation), the average tumor size in experimental and PBS groups was 53.21±10.45 mm^3^ and 59.12±8.45 mm^3^, respectively. Mice received NDV-based virotherapy and PBS on 20–23^th^ days of the experiment. The Fig 6 shows the increasing size of tumor node during the virotherapy and PBS injections, which may be related to the beginning of active tumor growth and inflammatory response to mechanical damage of tumor by injections. However, NDV/Altai/pigeon/770/2011-based virotherapy resulted in significant inhibition of tumor node growth throughout the experiment compared to control group. As soon as on 5^th^ day after virotherapy (28^th^ day of experiment), the average tumor size in the experimental group was 2.5 times less than in the control group (175.27±40.38 mm^3^ and 439.43±35.62 mm^3^, respectively). On 10^th^ day (33^rd^ day of the experiment), there was a 4.5-fold difference in the average size of tumor nodes in the experimental and control groups (of 122.01±32.38 mm^3^ and 560.37±53.42 mm^3^, respectively), and by the end of the experiment (15^th^ day after the course of virotherapy and 38^th^ day of the experiment), there was a 4.2-fold difference (247.70±63.43 mm^3^ and 1032.72±100.38 mm^3^, respectively) (Fig 7) (P <0.05).

#### NDV induces significant tumor necrosis in non-small cell lung cancer *in vivo*

**Fig 8:**
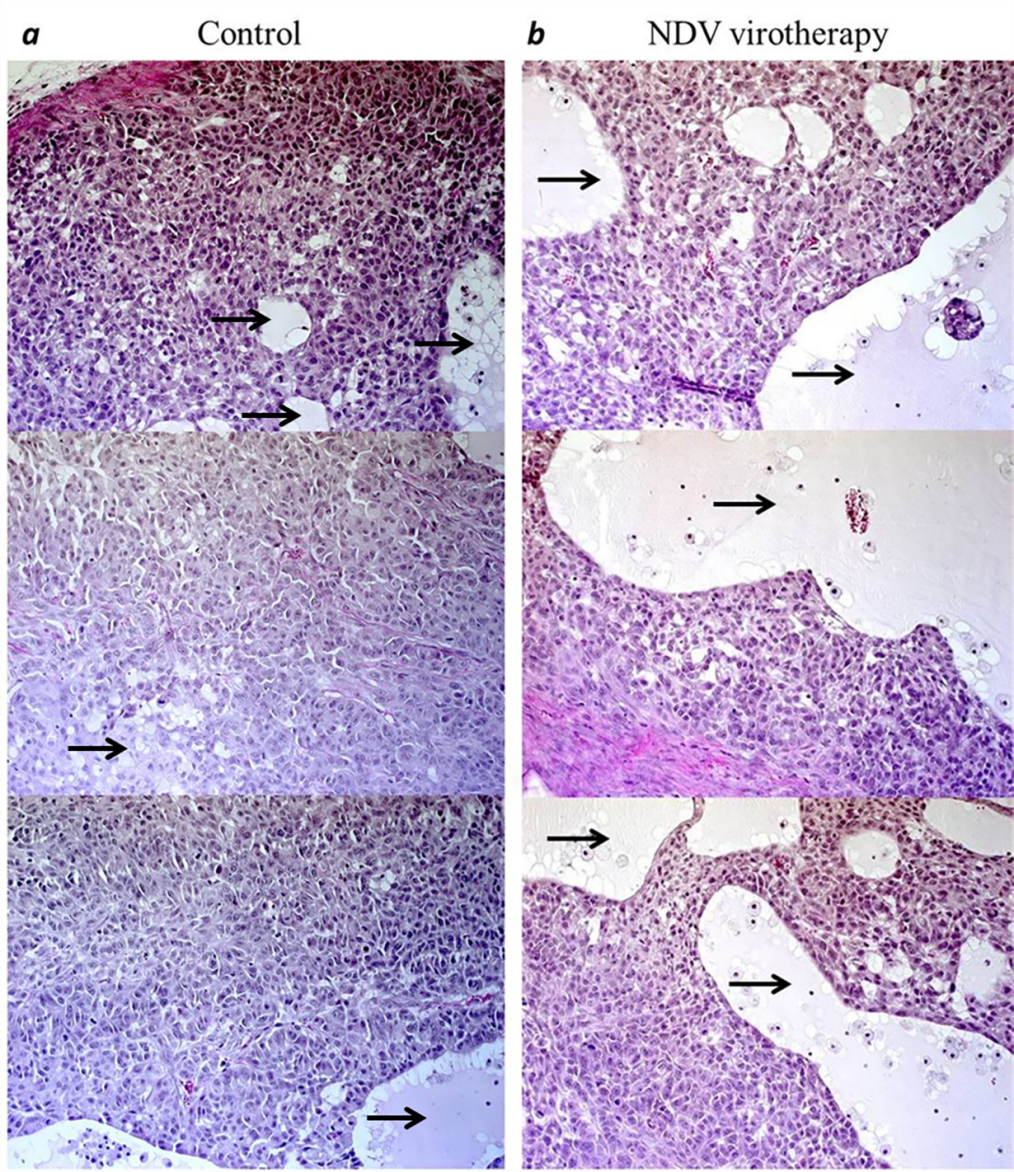
NDV treatment results in tumor necrosis in non-small-cell lung carcinoma–bearing mice. Male SCID mice bearing subcutaneous human non-small-cell lung carcinoma A549 nodules were infected with NDV/Altai/pigeon/70/2011 (7 lgTCID50/100μl) in series of intratumoral injections (one injection/day during 4 days). Mice (n = 5) were sacrificed 5, 10 and 15 days post-infection. Tumor sections were stained with hematoxylin and eosin (H&E) and microscopically analyzed for tumor necrosis. Massive sites of destructive necrosis on the 10^th^ day after virotherapy (a) vs tumor tissue of the control group of mice, receiving PBS injections (b). Areas of necrosis are denoted by arrows. Magnification ×20.

To determine the oncolytic potential of NDV/Altai/pigeon/770/2011 *in vivo*, we sacrificed animals at various time points after virotherapy (5, 10, and 15 days (28, 33 and 38 days of tumor growth, respectively)) and measured tumor virotherapy response and structural changes in tumor tissue. Histological examination of tumor node sections demonstrated increasing tumor volume and hemorrhages. The tumor nodes were restricted to connective tissue capsule. By 5^th^ day post-therapy, there were almost no necrotic areas in tumor tissue. Necrotic changes appeared later. Morphometric analysis of necrotic areas revealed increasing tumor necrosis, which peaked on 10^th^ day after therapy (Fig 8). On 10^th^ and 15^th^ days, necrotic areas with destructive swelling were often found. Structurally, destructive edema sites and necrotic foci accounted for 54.13% and 73.89% of tumor bulk density on 10^th^ and 15^th^ day after virotherapy, respectively.

## Discussion

NDVs are not pathogenic for mammals and have natural oncolytic properties, which makes them promising anticancer agents. Wild-type NDV strains are safer and more cost-effective than modified recombinant strains requiring additional measures for genetic control of viral products. Promising *in vitro* and *in vivo* findings on several attenuated wild-type NDV strains such as MTH-68/H [31] and HUJ [32, 33] were followed by encouraging results of clinical trials [34–36].

Here we investigate the oncolytic properties of 44 wild-type NDV isolates obtained from migratory birds in Russia in Western Siberia and the Far East from 2008 to 2014. Most of the viruses were isolated from *Anseriformes* and proved avirulent and lentogenic except one velogenic and several mesogenic isolates including NDV/Altai/pigeon/770/2011 [28].

MTT-based viability testing of tumor cells after incubation with NDV isolates demonstrated the oncolytic properties of the isolates in cell lines of different etiology and histogenesis. Tumor cell lines had different sensitivities to NDVs. The following cell lines were most sensitive: non-small-cell lung cancer A549 cells (17 isolates had anticancer effect) and cervical cancer HeLa cells (11 isolates had anticancer effect). Mammary adenocarcinoma MCF7 cells were least sensitive, and cell viability after incubation with the viruses remained high.

Among the 44 wild-type NDV isolates, tested for oncolytic potential in the present work, 7 isolates demonstrated the most pronounced oncolytic capacity in all human tumor cell lines–NDV/mallard/Amur/264/2009 (GenBank accession no. KX352835), NDV/teal/Novosibirsk_region/320/2010 (GenBank accession no.KX352836) and NDV/Novosibirsk/garganey/321/2010 and NDV/Novosibirsk/garganey/373/2010 and NDV/Altai/pigeon/770/2011 (GenBank accession no.KJ920204), NDV/Yakutiya/mallard/852/2011 (GenBank accession no.KJ920203) and NDV/Novosibirsk/shoveler/776/2010 (Fig 4).

The oncolytic properties of natural NDV isolates were dose-dependant. The viability of tumor cell lines decreased averagely by 5–10% with increasing virus dose (from 2 to 16 HAU per 10.000 cells). The oncolytic effect of the virus was independent from the etiology of tumor cells and was the same for colorectal cancer, cervical cancer or non-small-cell lung carcinoma.

However, specific NDV strains demonstrated different oncolytic properties with specific tumor cell lines. Several NDV strains were effective against one cell line and failed to eliminate others. For example, NDV/Yakutiya/mallard/852/2011 and NDV/teal/Novosibirsk_region/320/2010 were effective against A549 cells but had a poor performance with HeLa and HCT116 cells.

Oncolytic potential of all the 7 isolates is slightly different in relation to different tumor cell lines, however, the most pronounced antitumor effect observed for all studied cell lines, was observed for strain NDV/Altai/pigeon/770/2011 –from 20 to 40% of viable cells HCT116, A549 and HELA after incubation with virus dose 16 HAU per 10.000 cell, and about 80% for the resistant lines MCF7.

Numerous studies focused on investigations of mechanisms of viral-induced death in tumor cells. There is data that NDV can trigger not only apoptosis in the transformed cells, but also autophagy [37] and necrosis [15]. In the A549 cells, when infected with strains of different virulence (LaSota—lentogenic, Beaudette C—mesogenic, FMW—velogenic), activation of caspase-9 and caspase-8 was shown, at the same time interval and regardless of the pathotype of strain [38]. There is also evidence of the activation of the MARK signaling pathway during NDV-induced apoptosis in A549 cells, in which pathways are activated through ERK, JNK and p38 kinases, but the activation of p38-MAPK kinase plays a major role, regardless of the pathogenesis of the NDV strain [38]. Apoptosis can be induced by the matrix (M) protein of the Newcastle disease virus, located on the inner surface of the viral envelope. The proapoptotic effect of the M protein was demonstrated by the infection of the HeLa [39] and HCT116 and HT29 [40] cells with the Malaysian strain AF2240. The Newcastle disease virus can lead to the destruction of tumor cells by the mechanism of necrotic death [14, 15].

For a more detailed study of differences in the implementation of the oncolytic properties of natural isolates NDV further work is needed to explore possible differences in the mechanisms of penetration of viruses in tumor cells of different etiology and histogenesis, replication and release of infectious progeny, to evaluate the influence of genetic alterations of tumor cells on the program of oncolytic properties in virotherapy. For example, it was shown that the Ras-pathways activation is required for a successful replication of NDV, namely, its branches, related small GTP-Rac1 basics [41]. However, in the present study, despite the activation of Ras-pathways in the cells of tumor lines MCF7 (amplification of the Her), the implementation of anticancer potential in this line was not fully achieved with any studied strain.

In next part of study we have focused our attention on the ability of the virus to suppress tumor growth *in vivo*. To evaluate the effect of the virotherapy on the tumor progression and on the changes in the structure of tumor tissue after the viral treatment, we investigated the direct oncolytic influence of the course of intratumoral injections on the xenograft model of human non-small cell lung A549 carcinoma. Referring to the experience of carrying out the *in vivo* experiments described in the scientific literature on different tumor models and different strains of viruses, an amount of A549 carcinoma cells, dose of virus, volume of injection and method of administration were selected.

As was described in studies, virulent (mesogenic and velogenic) strains are able to induce more powerful apoptotic response in tumor cells and at very early stages of virus-cell interaction, in contrast to apatogenic strains and strains with reduced virulence [38, 42]. To choose one oncolytic strain to carry out in vivo experiment we decided to use mesogenic NDV/Altai/pigeon/770/2011. The usage of much more virulent strains in virotherapy can be more effective strategy for successful tumor treatment. For example, Ghrici M. et al. showed activation of caspase-8 already in 2 hours after infection of MCF7 cells with strain AF2240, in contrast to the data presented by Elankumaran S. et al. [43] and Ravindra P. [44], where activation of caspase-8 was demonstrated only after 48 and 24 hours, respectively. Such a temporal spread of caspase-8 activation could be associated not with the difference in the order of activation of the external and internal pathways of apoptosis, but probably with the dependence of the rate of apoptosis on the virulence of the strain. This time difference in activation of death program can be important for suppression tumor progression.

According to the analysis of the growth dynamics of the tumor node, we showed that in the control group of mice receiving injections of the PBS, the tumor process developed and progressed, while in the group that received virotherapy with NDV/Altai/pigeon/770/2011, the tumor growth rate decreased. Moreover, virus contributed to a decline of tumor growth at the initial stage after virotherapy (day 5) with an increase of the antitumor effect by 10 days. It is worth noting that, at the end of the experiment, there is an insignificant increase in the average volume of the tumor node. However, this growth is insignificant in comparison with the growth rates in the control group.

Tumor nodes from animals treated with virotherapy were much denser than the nodes from the control group of animals. These results suggest that there is the prevalence of fibrosis in experimental tumors after virotherapy. However, such a relationship was not revealed, because the bulk density of the connective tissue of experimental tumors was higher only on the 5^th^ day. At subsequent time points (10 and 15 days), the bulk density of the connective tissue was more than 3 times lower than in the control.

On the other hand, the dynamics of changes in the bulk density of connective tissue could indirectly suggest the increase in the volume density of necrosis with areas of destructive swelling, the numerical density of which increases from 5 to 15 days. With the intensification of necrotic processes, the volume density of the connective tissue also decreases. However, it should be noted that necrotic processes in the control group receiving injections of the PBS also increase in dynamics, that is probably due to the rapid growth of the tumor node and the formation of foci of ischemia.

Despite the fact that necrosis is considered to be an unfavorable development of destructive changes in tissue, necrosis can also pass under the control of cellular signaling pathways, being, like apoptosis, a process of regulated cell death. I*n vivo* study with recombinant rNDV/F3aa (L289A) showed that morphometric analysis in the tumor tissue of hepatocellular carcinoma after NDV administration through the hepatic artery revealed the increase of necrotic changes in tumor tissue. However histological studies of healthy tissue surrounding the tumor showed no hepatotoxicity—there was no syncytium formation (typical for NDV infection) in the surrounding tissue and no inflammatory infiltrates [15].

Obviously, the above results suggest the prospect of using the virus of Newcastle disease as an antitumor agent affecting tumor cells and tissues, causing their death. The data obtained suggest the NDV/Altai/pigeon/770/2011 strain for further investigation as an oncolytic agent whose antitumor mechanism of action requires further research.

In summary, we have characterized the ability of wild-type Newcastle disease virus to demonstrate strong oncolytic effects in direct lysis of human tumor cell lines of various histogenesis. Normal PBMC culture seems to be resistant. The efficacy of virotherapy of subcutaneous non-small cell lung A549 carcinoma in SCID-mice was demonstrated. Intratumoral course of NDV leads to inhibition of tumor progression, which is reflected in the dynamics of tumor nodule size by direct lytic effect bypassing the activation of immune system. Further work is necessary to investigate mechanisms of virus-induced cell death and the wild-type NDV/Altai/pigeon/770/2011 as the anticancer agent.

## Conclusion

We demonstrate possibility to use wild-type Newcastle disease virus isolated from birds in approach for cancer therapy. There may be lots of different recombinant oncolytic strains under investigation but we believe that it is important to propose a perspective for study the wild-type NDV strains for developing oncolytic anticancer agent. Based on the our results, such strains can be promising biological agents for future cancer therapy.

## Materials and methods

### Viruses

Newcastle disease virus isolates were gathered from wild birds within their migratory routes in the territory of Siberia and the Far East, Russia (Table 1). Cloacal and feather swabs were collected from August to October in 2008–2011 and 2014 in eight administrative regions of the Russian Federation: the Altai Territory, the Novosibirsk Region (Chany Lake, Western Siberia), the Republic of Tyva (Eastern Siberia) and the Amur Region, the Kamchatka Territory, the Republic of Sakha (Yakutia), the Sakhalin Region Far East) and one sample was isolated in the territory of the Republic of Adygea (Southern Federal District). The activities carried out using specimens were approved by the Committee on Biomedical Ethics of Research Institute of Experimental and Clinical Medicine, Novosibirsk (Permit Number: 15). The experiments were conducted in Laboratory of experimental modelling and pathogenesis of infectious diseases, Research Institute of Experimental and Clinical Medicine, Novosibirsk, Russia.

**Table 1:** Wild-type Newcastle disease virus isolates, gathered from migratory birds in Russia.

№	NDV isolates	Titer HA^a^	Viral pathotype^b^
1	NDV/Altai/garganey/49/2008	32	avirulent
2	NDV/Altai/gadwall/66/2008	16	avirulent
3	NDV/Amur/grasswarbler/267/2008	16	avirulent
4	NDV/Novosibirsk/mallard/718/2008	256	lentogenic
5	NDV/Novosibirsk/shoveler/738/2008	16	avirulent
6	NDV/Novosibirsk/garganey/753/2008	128	avirulent
7	NDV/Novosibirsk/garganey/767/2008	16	avirulent
8	NDV/Novosibirsk/garganey/769/2008	256	avirulent
9	NDV/Adygea/duck/12/2008	256	velogenic
10	NDV/Novosibirsk/shoveler/776/2008	256	avirulent
11	NDV/Altai/garganey/564/2009	16	avirulent
12	NDV/mallard/Amur/264/2009	256	lentogenic
13	NDV/Kamchatka/gull/12/2009	128	avirulent
14	NDV/Kamchatka/gull/528/2009	16	avirulent
15	NDV/Novosibirsk/garganey/428/2010	16	avirulent
16	NDV/Novosibirsk/shoveler/429/2010	64	avirulent
17	NDV/Novosibirsk/garganey/452/2009	16	avirulent
18	NDV/Novosibirsk/garganey/465/2009	32	avirulent
19	NDV/Novosibirsk/goosander/529/2009	16	avirulent
20	NDV/Novosibirsk/garganey/746/2009	32	avirulent
21	NDV/Novosibirsk/mallard/4112/2009	16	avirulent
22	NDV/Yakutiya/mallard/860/2009	256	lentogenic
23	NDV/Altai/pigeon/777/2010	128	mesogenic
24	NDV/Altai/mallard/784/2010	16	avirulent
25	NDV/Amur/garganey/922/2010	64	avirulent
26	NDV/Amur/dove/992/2010	16	avirulent
27	NDV/teal/Novosibirsk region/320/2010	256	lentogenic
28	NDV/Novosibirsk/garganey/321/2010	128	lentogenic
29	NDV/Novosibirsk/garganey/322/2010	8	avirulent
30	NDV/Novosibirsk/garganey/329/2010	128	avirulent
31	NDV/Novosibirsk/garganey/339/2010	128	avirulent
32	NDV/Novosibirsk/garganey/373/2010	32	avirulent
33	NDV/Novosibirsk/garganey/389/2010	128	avirulent
34	NDV/Novosibirsk/shoveler/396/2010	16	avirulent
35	NDV/Novosibirsk/gadwall/703/2010	8	avirulent
36	NDV/Novosibirsk/shoveler/945/2010	32	avirulent
37	NDV/Sakhalin/pintail/47/2010	8	avirulent
38	NDV/Sakhalin/widgeon/48/2010	32	avirulent
39	NDV/Sakhalin/garganey/52/2010	32	avirulent
40	NDV/Sakhalin/pintail/53/2010	256	avirulent
41	NDV/Altai/pigeon/770/2011	128	mesogenic
42	NDV/Yakutiya/mallard/852/2011	128	mesogenic
43	NDV/Novosibirsk/garganey/27/2014	128	lentogenic
44	NDV/Tyva/gull/14/2014	128	lentogenic

^a^—harvesting in ECE system, titer HA assay in HAU per 50 μl in virus contained allanoic liquid (HAU/50 μl), ECE–embryonated chicken egg.

^b^—based on both pathogenicity assays (MDT, ICPI) according to classification [47].

No specific permission was required for sample collection from wild birds killed by local hunters in compliance with the Russian Federation hunting laws. Also no permit was needed for sampling from wild birds captured alive during ringing activities in Biostations with collaboration with other research institute as part of the national avian influenza surveillance.

The original stocks were amplified in the first passage using 10-day-old embryonated eggs from non-vaccinated specific-pathogen free chicken according to the WHO recommendations [45]. After 72 hours post inoculation, the virus containing allantoic fluid was harvested and partially purified through 0.45 μm porous filters and stored at -80°C. The presence of Newcastle disease virus in the allantoic fluid was determined by the hemagglutination reaction and RT-PCR with specific primers [46]. Based on the obtained pathogenicity test results–mean death time (MDT) and intracerebral pathogenic index (ICPI), it can be concluded that the 44 NDV investigated isolates are avirulent, with the exception of the several lentogenic strains and virulent strains—NDV/Altai/pigeon/770/2011 [28], NDV/Altai/pigeon/777/2010 [29] and NDV/Yakutiya/mallard/852/2011 [27], which belongs to the group of mesogenic strains, and the strain NDV/Agydea/Duck/12/2008 [29], belonging to the group of highly pathogenic velogenic strains.

### Cell lines and culture conditions

We studied 4 human cancer cell lines of various etiologies. Human colon carcinoma cell line HCT116 was obtained from State Research Center of Virology and Biotechnology «Vector» (Novosibirsk region, Koltsovo, Russia), human breast adenocarcinoma cell line MCF7 was obtained from Russian collection of cell lines of vertebrates of the Institute of Cytology Russian Academy of Sciences (Saint-Petersburg, Russia), human epitheloid cervix carcinoma HeLa was kindly donated by Institute of Clinical and Experimental Lymphology (Novosibirsk, Russia), human non-small lung carcinoma cell line A549 was a kind gift from Belogorodstev S., Research institute of fundamental and clinical immunology (Novosibirsk, Russia). All cell lines were grown in Dulbecco’s modified eagle’s media (DMEM) (Gibco Inc., UK) with 1% antibiotic (penicillin, streptomycin, gentamicin) solution and 10% heat-inactivated fetal bovine serum (Gibco Inc., South America) and were maintained at 37°C in an incubator with 5% CO_2_.

Human peripheral blood mononuclear cells (PBMC) were selected as control healthy non-transformed cells. PBMCs were isolated from blood of conditionally healthy donors by the method of sedimentation in a ficoll density gradient. The blood sampling protocol was approved by the Committee on Biomedical Ethics of Research Institute of Experimental and Clinical Medicine, Novosibirsk (Permit Number: 15). Written informed consent was obtained prior to the study from the human volunteer. Peripheral blood mononuclear cells were cultured in RPMI medium with 10% heat-inactivated fetal bovine serum (Gibco Inc., South America) and with 1% antibiotic (penicillin, streptomycin, gentamicin) solution at 37°C and in an atmosphere with 5% CO_2_.

### Cytotoxicity assay

Tumor cells were plated at 3 × 10^4^ cell per well in 96-well plates in 0.2 ml of MEM supplemented with 1% heat-inactivated FBS with 1% antibiotic (penicillin, streptomycin, gentamicin) solution. After 24 hours, tumor cells were infected with NDV isolates at different doses– 2, 8 and 16 HAU per 10.000 cells in 1% FBS maintenance medium. After incubation with virus for 1 hour, virus was removed and 0.2 ml fresh maintenance medium was added. Mock cells were incubated with 1% FBS MEM medium. Cell viability was analyzed using MTT assays at time point 96 hours after NDV infection. The principle of the method is based on the ability of mitochondrial succinate dehydrogenase to transfer the water-soluble yellow MTT salt into violet crystals of formazan, which accumulate in the cytoplasm of cells. On the level of accumulation intensity of these crystals, one can judge the viability of cells in a cellular monolayer. First, cells were washed two times with Hanks solution. Stock MTT solution was prepared in concentration 5mg/ml in phosphate buffered saline (PBS). 10%-MTT working solution was prepared on the basis of a maintenance MEM medium. 0,1 ml of 10%-MTT was added to each well. Tumor cells were incubated with MTT for 4 hours at 37°C in an incubator with 5% CO_2_ and lysed in DMSO (0.15 ml/well) for 1 hour at room temperature in dark place to release crystals of formazan. These result in changing the color of medium and are due to activity. The optical density was measured at a wavelength of 540 nm and 630 nm (background) on a LonzaBiotek ELX808 Absorbance Microplate Reader (USA).

To evaluate safety for PBCM equal volumes of the cell suspension in the growth medium were distributed over the tubes. The cells were concentrated by centrifugation 1500g for 5 minutes, the supernatant was removed and incubated with a different dilution of the virus in 1%-FBS RPMI medium with 1% antibiotic from 2 to 32 HAU per 10.000 cells. The virus concentration was increased in 2-fold increments. Cells with the virus were incubated for 1 hour at 37°C in an incubator with 5% CO_2_, then the cells were concentrated by centrifugation 1500 g for 5 minutes, the supernatant was removed and cells were resuspended in fresh medium and then plated on 96-well plate at 10,000 cells in 100 μl per hole. Control tumor cells were incubated in 1%-FBS RPMI medium with antibiotics without virus for 1 hour.

Three days after infection of the cell lines with NDV isolates, an analysis of the viability of PBCM-cells was carried out using the commercially available CellTiter 96®AQueous One Solution Cell Proliferation Assay (Promega, USA). This reagent contains the tetrazolium compound MTS, which in living cells with enzymatic activity is reduced to formazan, staining the culture medium. To each well, 20 μl of reagent was added to 100 μl of the culture medium and incubated for 2 hours at 37°C in an atmosphere with 5% CO^2^. The optical density was measured at a wavelength of 490 nm.

The percentage of living cells was calculated by the formula:

$$\frac{E540-E630}{K540-K630}\times 100\%,$$ (1)

where E is the indices obtained from the virus infected wells, and K is the parameters of the control wells. All viral isolates were analyzed on each tumor cell line in triplicate. The results of measured cell viability are expressed as the percentage of surviving of infected cells versus uninfected mock cells which were considered to be 100% of viability.

### Animals and xenograft human non-small lung carcinoma model

Male 7-8-weeks-old SCID immunodeficient mice were purchased from Institute of Cytology and Genetics SB RAS and were housed under special conditions (in sterile, filtered cages) with food and water *ad libitum*. Animal studies were conducted at the Center for Genetic Resources of Laboratory Animals at the Institute of Cytology and Genetics, Siberian Branch, Russian Academy of Sciences.

The number of A549 non-small lung carcinoma cells injected to induce xenograft was chosen after trial experiments with different concentrations of cells. Concentration of 3.5 × 10^6^ A549 cells in 100 μl of PBS per mouse proved to be preferable in terms of the developmental and growth dynamics of the tumor node, which makes possible to use tumor-bearing animals in the experiment according to the rules of the bioethical commission. All efforts were made to minimize suffering. During the experiment with SCID-mice we did not find dead mice. Also no mouse was euthanized because of inactivity, significant weight loss, inappetence, piloerection and other symptoms. Experiments protocols were approved by the Committees on Biomedical Ethics of Research Institute of Experimental and Clinical Medicine, Novosibirsk (Permit Number: 15).

Mice were injected subcutaneously into the right flank with 3.5 × 10^6^ in 100 μl PBS. Subcutaneously solid tumor node is formed without metastasis. Tumor nodules became visible in place of the A549 on the 7th day among 100% of inoculated animals. The design of experiment ensured efficiency as it is possible with the object of obtaining consistent results with the minimum number of animals and the minimum degree of pain and suffering, according to the European convention for the protection of vertebrate animals used for experimental and other scientific purposes (Strasbourg, 18.III.1986). On 10^th^ day nodules began to measure metrically. The nodes reached the desired diameter (4 mm) for initiation of the course of virotherapy on 20^th^ day after inoculation of tumor cells. The dynamic of tumor growth allows to conduct a course of viral treatment and to observe the animals for 20 days after virotherapy.

### Newcastle disease virotherapy

Viral treatment was performed with NDV/Altai/pigeon/770/2011 strain. SCID mice were divided into three groups: group1 –control tumor-bearing animals receiving 4 viral dose injections every other day starting 20 day after inoculation of tumor cells. NDV was administrated intratumorally into solid node at a dose of 7 lgTCID50/100μl. Virus titers were determined as TCID50/ml on Vero cell line, obtained from State Research Center of Virology and Biotechnology «Vector» (Novosibirsk region, Koltsovo, Russia) [48]. Group 2—experimental tumor-bearing animals receiving 4 injections of 100 μl PBS every other day starting 20 day after inoculation of tumor cells. All volume of 100 μl was injected into the solid tumor by 2–3 several injections from different sides of tumor node to spread the virus into the tissue more effectively. Group 3 –control animals without tumor but receiving injections of the PBS instead of introducing tumor cells and injection of the virus subcutaneously.

On the 10^th^ after tumor cell inoculation, during virotherapy and after treatment mice were regularly weighed in 2–3 days and observed for tumor growth by measuring of dynamics of changes in the size of the tumor nodes. Five animals were randomly chosen for killing at each of the following time points: day after completion of therapy 5, 10, and 15 (28, 33 and 38 days of tumor growth, respectively). Animals from the control and experimental groups were removed from the experiment by dislocation of the cervical vertebrae. Upon killing, biomaterial of subcutaneous tumor was taken for histological diagnosis. Animals from the group 3 were observed during the whole time of the work and were withdrawn from the experiment on 38^th^ day of tumor growth.

Tumor growth was monitored by callipers measuring each 2–3 days from the first day of virotherapy till the end of experiment. The measurements of the length and width of the subcutaneous tumor node were calculated by the standard formula and the received tumor volume

$$V={\frac{\left(length)\times (width\right)}{2}}^{2}(mm^{3}).$$ (2)

The animals were placed under observation every 2 days with a check of changes in the state of motor activity, changes in body weight, skin and mucous membranes, food intake and water. Endpoint of the experiment and the last point of biomaterial sampling (38 days of tumor growth) was chosen in according with statement that tumor size must not exceed 15 mm (1.5 cm) at the largest diameter in an adult mouse, because larger tumor may include mobility restriction, the inability to access food and water, pressure on internal organs or sensitive regions of the body.

### Histology

In tumor-bearing animals subcutaneous tumor samples were harvested. Tumor tissue were fixed in 4% paraformaldehyde and paraffin embedded. Sections of 4 μm thickness were subjected to hematoxylin–eosin staining (H&E) for histological analysis and Van Gieson staining.

### Statistical analysis

For comparison of individual data points, the student t-test was applied to determine statistical significance. P values of <0.05 were considered statistically significant. The results of the evaluation of the viability of tumor cell lines are presented in the form of histograms in percent as the average relative values of the proportion of living cells on the fourth day after treatment with virus to the proportion of control cells not treated with virus, taking into account the standard deviation (mean relative value ± standard deviation). In animal experiment, dynamic of tumor growth is presented by graphic. Statistical data were obtained using Statistica 6.0 software.
